# Reports of Lunatic Asylums

**Published:** 1846-05-01

**Authors:** 


*Reports of Lunatic Asylums.*—We have been favored with a copy of the third annual report of the State Lunatic Asylum of Utica, and, also, the twenty-fifth annual report of the Bloomingdale Asylum, for which the medical superintendents of these institutions will please accept our thanks. We had prepared an article of some length suggested by their perusal, which the compositor declares must be omitted. We can only say that their reports afford gratifying evidence that the State of New-York is not backward in her efforts in behalf of this department of public philanthropy, and that there is abundant cause for congratulation that the managers have been able to secure the services of medical officers so eminently qualified to carry out the benevolent designs of the institutions under their charge.

